# Dr. S. C. Wilkerson

**Published:** 1898-01

**Authors:** 


### DR. S. C. WILKERSON.

Died, at his home, in Tuscaloosa, Alabama, October 15, 1897, Dr. Sim. Cornelius Wilkerson.

On the 25th of February, 1896, Dr. Wilkerson was stricken with paralysis, from which he never entirely recovered and was almost helpless until his death. He was born in

Foster's Settlement, Tuscaloosa county, Alabama, May 11, 1839. After a course at the University of Alabama he began the study of dentistry under the pupilage of Dr. E. H. Cogburn, in 1857, attended a course of lectures at the Baltimore College of Dental Surgery (session 1858-9), and began the practice of his profession in Tuscaloosa the following autumn. At the opening of the Civil War he was a member of the "Warrior Guards" of Tuscaloosa, with which company he was called into the Confederate service as a private. In the early part of 1861 he returned to his native county and, with three others, organized a volunteer company, of which he was elected first lieutenant, and this company formed a part of the eighteenth Alabama Infantry. He was promoted to the rank of captain early in the war, and was captured at the battle of Missionary Ridge, after being slightly wounded. He was a prisoner of war on Johnson's Island about eighteen months, until the close of the war, when he returned to Tuscaloosa, and resumed his practice August 5, 1865, which was at once successful and lucrative. He retired in 1870 to engage in business in New Orleans, La., but again resumed his practice in Tuscaloosa in 1879, and continued there in active professional work until he retired, which he did shortly before his attack of paralysis. He was a member of the Alabama Dental Association, which body elected him a delegate to the American Dental Association and the National Dental Association sessions, which convened in Boston and New York City, respectively, in 1880, neither of which meetings he could attend. During his course of a large practice he had several dental pupils who afterward attended the Baltimore College of dental Surgery. During the war he married Miss Nannie Bryson, an accomplished lady of Macon, Mississippi, who survives him.

Early in life he joined the Baptist Church, and continued a faithful member until his death. He was an affectionate husband, son, and brother; fond of his profession, a good and active citizen, and highly esteemed by all who knew him. —*Dental Cosmos*.

